# Divalent magnesium restores cytoskeletal storage lesions in cold-stored platelet concentrates

**DOI:** 10.1038/s41598-022-10231-x

**Published:** 2022-04-14

**Authors:** Konstanze Aurich, Jan Wesche, Martin Ulbricht, Oliver Otto, Andreas Greinacher, Raghavendra Palankar

**Affiliations:** 1 Institut für Transfusionsmedizin, Universitätsmedizin Greifswald, Sauerbruchstraße, 17475 Greifswald, Germany; 2 Zentrum Für Innovationskompetenz: Humorale Immunreaktionen Bei Kardiovaskulären Erkrankungen, Universität Greifswald, Fleischmannstr. 42, Greifswald, Germany; 3 Present Address: Institut Für Pharmazie, Universität Greifswald, Friedrich-Ludwig-Jahn-Straße 17, Greifswald, Germany

**Keywords:** Medical research, Translational research

## Abstract

Cold storage of platelet concentrates (PC) has become attractive due to the reduced risk of bacterial proliferation, but in vivo circulation time of cold-stored platelets is reduced. Ca^2+^ release from storage organelles and higher activity of Ca^2+^ pumps at temperatures < 15 °C triggers cytoskeleton changes. This is suppressed by Mg^2+^ addition, avoiding a shift in Ca^2+^ hemostasis and cytoskeletal alterations. We report on the impact of 2–10 mM Mg^2+^ on cytoskeleton alterations of platelets from PC stored at room temperature (RT) or 4 °C in additive solution (PAS), 30% plasma. Deformation of platelets was assessed by real-time deformability cytometry (RT-DC), a method for biomechanical cell characterization. Deformation was strongly affected by storage at 4 °C and preserved by Mg^2+^ addition ≥ 4 mM Mg^2+^ (mean ± SD of median deformation 4 °C vs. 4 °C + 10 mM Mg^2+^ 0.073 ± 0.021 vs. 0.118 ± 0.023, *p* < 0.01; n = 6, day 7). These results were confirmed by immunofluorescence microscopy, showing that Mg^2+^  ≥ 4 mM prevents 4 °C storage induced cytoskeletal structure lesion. Standard in vitro platelet function tests showed minor differences between RT and cold-stored platelets. Hypotonic shock response was not significantly different between RT stored (56.38 ± 29.36%) and cold-stored platelets with (55.22 ± 11.16%) or without magnesium (45.65 ± 11.59%; p = 0.042, all n = 6, day 1). CD62P expression and platelet aggregation response were similar between RT and 4 °C stored platelets, with minor changes in the presence of higher Mg^2+^ concentrations. In conclusion, increasing Mg^2+^ up to 10 mM in PAS counteracts 4 °C storage lesions in platelets, maintains platelet cytoskeletal integrity and biomechanical properties comparable to RT stored platelets.

## Introduction

Platelet transfusions are a mainstay of treatment in patients with hypoproliferative thrombocytopenia or major hemorrhage^1^. Platelet concentrates are stored at room temperature (RT), which preserves in vivo platelet survival and recovery. However, this increases the risk for bacterial growth. Cold storage of platelet concentrates at 4 °C reduces the risk of bacterial proliferation^1^. Recent studies have shown that cold storage maintains and even improves some in vitro platelet functions in comparison to storage at RT^2,3^. However, the in vivo circulation time/life span of cold-stored platelets is considerably reduced^4,5^. Loss of glycoprotein Ibα (GPIbα) due to increased metalloproteinase ADAM17 activity and glycan modifications are thought to play a role^6,7^. Cleavage of terminal sialic acid and clustering of exposed β-N-acetyglucosamines of GPIbα^5^ exposes β-galactose on the platelet surface. This is recognized by the Ashwell Morell receptor of hepatocytes or liver macrophages, which then phagocytose platelets^8,9^. Furthermore, temperatures below 15 °C induce divalent calcium (Ca^2+^) release from platelet storage organelles, and higher activity of Ca^2+^ pumps both results in increased intracellular Ca^2+^^4^. This triggers signaling cascades leading to cytoskeleton rearrangements and platelet shape change^10^, thought to mainly occur as a result of increased activity of the Ca^2+^ dependent cysteine protease calpain, which cleaves several of the cytoskeletal proteins and protein kinase C^11^. The Ca^2+^ pump transient receptor potential melastatin-like 7 channel (TRPM7) also regulates magnesium (Mg^2+^) influx into platelets. Mg^2+^ dependent TRPM7 kinase phosphorylates non-muscle myosin IIA (NMMIIA) that mediates the contractility of the actin cytoskeleton^12–14^. Mg^2+^ reverses cold-induced intracellular Ca^2+^ increase and inhibits consecutive cytoskeletal alterations^12^. Cytoskeletal integrity thus may serve as a sensitive read-out for cold storage induced platelet cytoskeleton lesions. To assess cytoskeleton-dependent biomechanical properties of platelets, several biophysical methods are available^15^. We used real-time deformability cytometry (RT-DC), for mechanical characterization of platelets with high throughput of up to 1,000 cells per second to evaluate the impact of cold storage on platelet cytoskeleton^16–19^.

Here, we report that the addition of up to 10 mM Mg^2+^ to platelets stored in platelet additive solution (PAS) plus 30% plasma prevents cytoskeleton alterations during long-term cold storage at 4 °C, while maintaining platelet function. In addition, we present a new ex vivo quality control approach to assess platelet concentrates in a whole blood matrix.

## Results

### Leukocyte and platelet depleted whole blood as a matrix to assess the function of platelet concentrates

We developed a method, which allows ex vivo quality control of PC under whole blood conditions. Leukocytes and platelets are considerably reduced by inline filtration of whole blood (Table 1). This provides a near platelet-free whole blood matrix. This platelet-depleted whole blood can be spiked with platelets of interest to assess platelet function in the presence of red cells and plasma proteins.

**Table 1:** Cell counts before and after filtration of whole blood by LEUCOFLEX® LXT filter (mean ± standard deviation, n = 3).

Cell count	Whole blood before filtration	Whole blood after filtration
Red blood cell (× 10^12^/L)	5.3 ± 0.3	6.5 ± 2.2
Leukocytes (× 10^9^/L)	5.8 ± 1.1	0
Platelets (× 10^9^/L)	247.3 ± 34.6	1.9 ± 0.2

### Magnesium does not adversely affect platelet function in vitro

We prepared PC in 70% PAS-E storage buffer (which contains 2 mM Mg^2+^) plus 30% plasma and added 2, 4, 6, and 8 mM Mg^2+^ before storage (final Mg^2+^ concentrations 2, 4, 6, 8, 10 mM). The additional magnesium did not exceed the physiological tonicity as it results in a maximum osmolarity of 319 mOsm/L (Supplementary Fig. S1).

**Figure 1:**
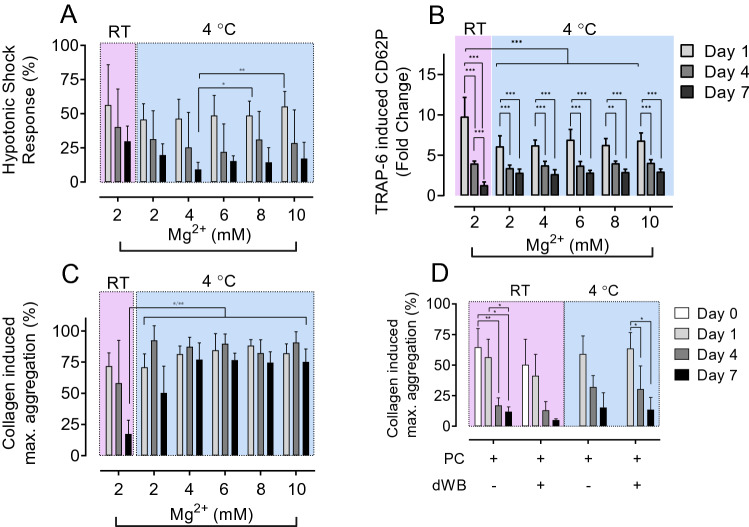
(**A**) Hypotonic shock response, (**B**) fold increase of CD62P mean fluorescence intensity after 20 µM TRAP-6 addition in comparison to buffer-control, and (**C**) maximum aggregation after 8 µg/mL collagen I addition to platelets from PC stored at RT + 2 mM Mg^2+^, 4 °C + 2, 4, 6, 8 or 10 mM Mg^2+^ (n = 6, mean ± SD). (**D**) Maximum aggregation after 8 µg/mL collagen I addition to platelets from PC stored at RT or 4 °C either subsequently added to platelet depleted, tempered whole blood (dWB) or not (n = 6, mean ± SD). *PC* platelet concentrate, *RT* room temperature, *Mg*^*2*+^ magnesium, *SD* standard deviation, *p < 0.05, **p < 0.01, ***p < 0.001, Statistical analysis is performed using one way ANOVA with multiple comparisions.

Hypotonic shock response did not differ substantially between the different storage conditions or Mg^2+^ concentrations (Fig. 1A). Also, increasing Mg^2+^ concentrations did not alter platelet reactivity to TRAP-6, as measured by CD62P expression, after 4 °C storage (Fig. 1B), which was reduced in comparison to RT stored platelets at day 1: fold change RT (2 mM Mg^2+^) 9.8 ± 2.4 vs. 4 °C (2 mM Mg^2+^) 6.1 ± 1.3 (*p* < 0.001). Thereafter the differences were minor between both storage conditions. Significant differences in CD62P expression occur between storage days 1 and 4 or 7 independently of the storage conditions.

In contrast to TRAP-6, platelet response to collagen (8 µg/mL) was better preserved in cold stored platelets after 7 days of storage (day 7: RT (2 mM Mg^2+^) maximum aggregation 22.54 ± 9.61% vs 4 °C (2 mM Mg^2+^) 45.96 ± 19.24%, n = 6, *p* < 0.01), especially at higher magnesium concentrations after 7 days of storage (4 °C + 10 mM Mg^2+^: maximum aggregation 77.97 ± 5.87%, n = 6, *p* < 0.01, Fig. 1C).

Spiking leukocyte and platelet depleted whole blood with platelets from differently stored PC and rewarming them at 37 °C for 1 h did not substantially change the aggregation response to collagen compared to platelets measured in PRP (Fig. 1D).

### Magnesium prevents platelet cytoskeleton storage lesions

**Figure 2:**
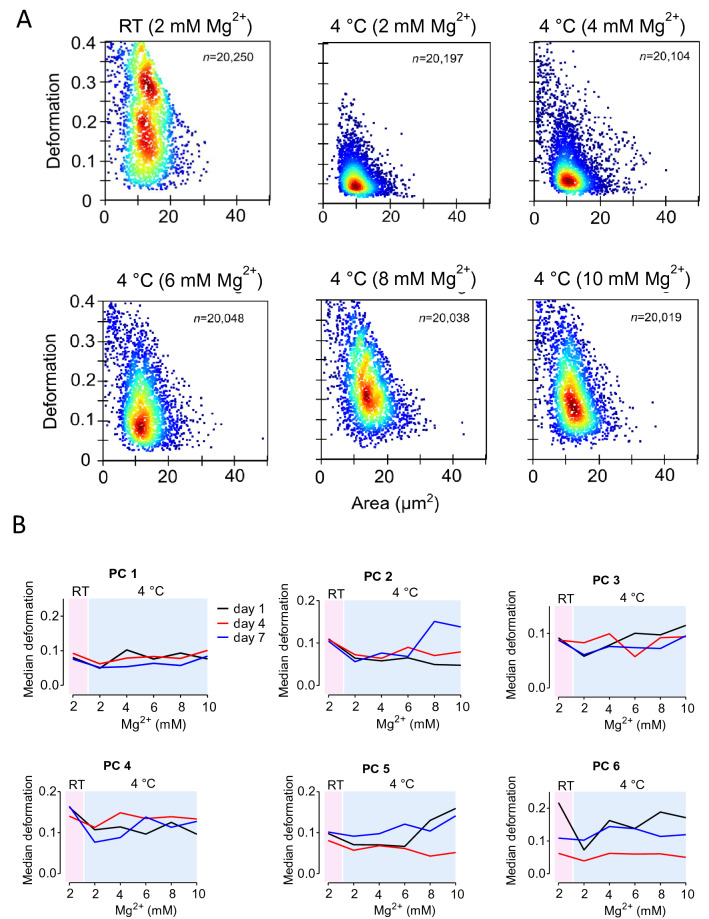
(**A**) Deformation vs cell area scatterplots determined by RT-DC of platelets from one PC stored for 7 days under different conditions at RT + 2 mM Mg^2+^, 4 °C + 2, 4, 6, 8 or 10 mM Mg^2+^. (**B**) Median deformation of platelets in platelet concentrates (PC, n = 6) for each magnesium concentration and storage temperature are given for day 1 (black), day 4 (red) and day 7 (blue), n ≥ 20,000 platelets. *RT* room temperature, *Mg*^*2*+^ magnesium.

The deformation to area scatterplots by RT-DC showed an apparent decrease in deformation and cell area of platelets stored at 4 °C (Fig. 2A). This was primarily prevented by increasing concentrations of Mg^2+^. For all six PC tested, median deformation (n ≥ 20,000 single platelets) decreased due to storage at 4 °C and generally increased with Mg^2+^ addition (Fig. 2B). This effect was particularly pronounced on storage day 7.

**Figure 3:**
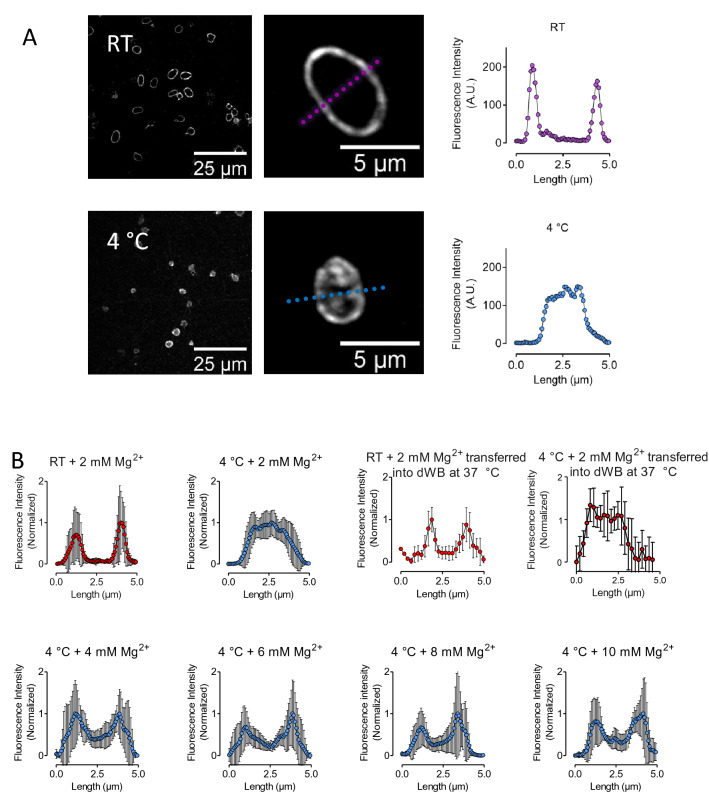
(**A**) Fluorescence image and intensity of stained α-tubulin along an axis of the cross-section of platelets from PC stored at RT or 4 °C. (**B**) Normalized fluorescence intensity of stained α-tubulin along an axis of the cross-section of platelets stored for 24 h under different conditions (n = 10, mean ± standard deviation). α-tubulin was stained by mouse anti-human α-tubulin IgG + secondary donkey anti-mouse IgG Alexa Flour 568. *RT* room temperature, *Mg*^*2+*^ magnesium, *dWB* platelet depleted whole blood.

Next, we assessed the integrity and reorganization of the marginal band tubulin ring. After storage at RT the tubulin ring is visible, both in the microscopic image and by the separated peaks of the cross-sectional fluorescence signals (Fig. 3A). Storing platelets at 4 °C induces disorganization of tubulin and distribution of the fluorescence signal throughout the cell (Fig. 3A bottom, Fig. 3B top left). Rewarming of platelets from cold-stored PCs in platelet-depleted whole blood has no consequences on the disorganization of the tubulin ring at 4 °C (Fig. 3B top right). Additional Mg^2+^ prevented tubulin depolymerization with the, most substantial effects at 8 and 10 mM Mg^2+^ (Fig. 3B bottom).

### Magnesium addition does not impair desialylation of platelets during cold storage

**Figure 4:**
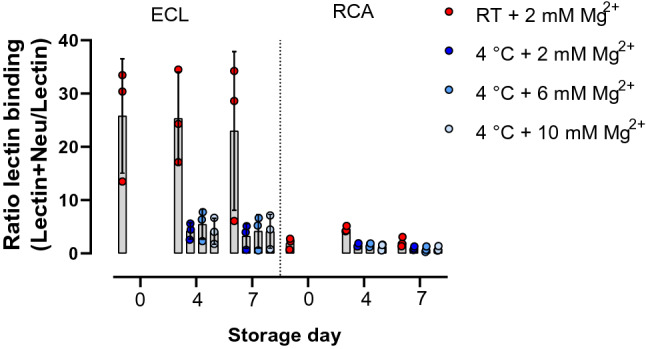
Impact of Mg^2+^ addition to desialylation of platelets. Binding of fluorescently labeled *Erythrina cristagalli* lectin (ECL) and *Ricinus communis* agglutinin (RCA) to platelet surface glycoproteins of RT and cold-stored platelets with or without Mg^2+^ in the presence and absence of sialidase during 7 days storage. *RT* room temperature, *Mg*^*2*+^ magnesium. n = 3, mean + standard deviation.

Cold storage initialized desialylation of platelet surface, resulting in increased phagocytosis of platelets by hepatocytes via the Ashwell-Morell receptor. We determined the binding of two different lectins, *Erythrina cristagalli* lectin (ECL) and *Ricinus communis* lectin agglutinin (RCA), to desialylated galactose and β-N-acetyl glucosamine (GlcNAc) residues on the surface of RT and cold-stored platelets with or without additional magnesium. Lectin binding is reduced due to cold storage but did not change with increasing magnesium concentration (Fig. 4).

## Discussion

In this study, we provide evidence that increasing the Mg^2+^ concentration of the storage medium up to 10 mM prevents platelet cytoskeleton storage lesions during storage at 4 °C. Otherwise, platelets storage at 4 °C leads to shape change, spherocytosis, and decreased mean platelet volume^4,10^. Recently, it has been shown that the platelet shape change, which is driven by the cytoskeleton, is mainly caused by two processes: (i) increased activity of calpain, a Ca^2+^ dependent cysteine protease, which cleaves platelet actin leading to rigid, stiff cells^20^ and (ii) upregulated NMMIIA activity, a motor protein mediating contractility of the platelet actin cytoskeleton^12^. During cold storage, intracellular Ca^2+^ increases due to active transport or passive Ca^2+^ leakage from platelet organelles and reduced Ca^2+^ pump activity leading to a slow accumulation of Ca^2+^^10^. Subsequently, store-operated Ca^2+^ entry (SOCE) and receptor-operated Ca^2+^ entry regulate further Ca^2+^ increase, which regulates platelet integrin αIIbβ3 activation by calpain and platelet degranulation^11,21,22^. TRPM7 channel is the key channel controlling Mg^2+^ and Ca^2+^ exchange in platelets^12^. TRPM 7 kinase activity is mediated by Mg^2+^ and directly affects intracellular Ca^2+^ concentration via SOCE, phospholipase C and γ^2^^13^. As a consequence Mg^2+^ supplementation counteracts intracellular Ca^2+^ increase, which prevents calpain activation. Furthermore, NMMIIA activity can be inhibited by enhanced intracellular Mg^2+^ concentrations, which increases adenine diphosphate (ADP) release, followed by induction of NMMIIA C-terminus phosphorylation^20^. This interplay between Ca^2+^ and Mg^2+^ is key to our concept to prevent alterations of the platelet cytoskeleton during cold storage by Mg^2+^ supplementation.

We also demonstrate that cold-induced cytoskeletal disorganization of platelets during storage at 4 °C is prevented by the addition of Mg^2+^ into the storage media. This we show by two independent methods: First by confocal laser scanning microscopy of platelet cytoskeletal proteins. However, immunofluorescence studies of platelets are low throughput and are at risk of a selection bias. Second by RT-DC, which we have recently introduced as a high throughput method to assess platelet biomechanics^23^. RT-DC allows measuring thousands of platelets within minutes and is the first method providing an unbiased, comprehensive analysis of the biomechanics of platelets in platelet concentrates. Consistent with our immunofluorescent studies, RT-DC shows preservation of platelet deformation at 4 °C by the addition of Mg^2+^.

The main reason for the reduced platelet survival after cold storage might be desialylation of platelet glycoproteins resulting in increased phagocytosis by hepatocytes. We showed that increased Mg^2+^ addition does not reduce desialylation during cold storage of platelets in vitro. It is more likely that magnesium affects platelet survival by other mechanisms. The exact molecular mechanisms of how increased Mg^2+^ concentrations prevent changes of the platelet cytoskeleton are still only partly understood. One proposed mechanism is by inhibiting the cold-induced increase in intracellular Ca^2+^ concentrations without activating calpain. Another potential mechanism might be the reduction of intracellular Ca^2+^ release after activation of GPIb. The binding of von Willebrand factor (vWF) to a mechanosensitive domain of GP Ibα triggers outside-in mechanotransduction signals in platelets, leading to intracellular Ca^2+^ release^24^. Platelet concentrates have to be stored under agitation that platelets can move along the gas permeable plastic membrane of the storage bag. This allows the release of CO_2_ and uptake of O_2_. Likely the shear stress on the plastic membrane of the platelet concentrate storage bag unfolds vWF, which then binds GPIb mediating mechano-signaling, which finally leads to an increase in plasmatic Ca^2+^ concentrations. In line with this hypothesis, blocking the vWF-GPIbα interaction by the peptide OS1 that blocks vWF binding to GPIbα, resulted in increased post-transfusion recovery and survival of cold-stored platelets in mice^25^.

The currently used PAS-E already contains 2 mM Mg^2+^. However, this concentration is not sufficient to prevent platelet storage lesions of the cytoskeleton. Our study shows that at least Mg^2+^ concentrations of 4 mM are required to prevent platelet cytoskeleton storage lesions. Increasing ion concentrations raise the issue of changing the osmolarity of the storage solution. However, at 10 mM Mg^2+^ PAS, osmolarity increased only by 4%, which is obviously well tolerated by platelets.

These concentrations are safe for the transfusion recipient. Mg^2+^ had been used extensively in obstetrics to control uterus contractions in concentrations up to 100 mmol given intravenously. This is 40-fold more than the Mg^2+^ dose given with a standard platelet concentrate^26^. Even the high concentrations given in obstetric patients have shown to be safe regarding the cardiovascular system and liver disease^27–29^.

Mg^2+^ addition to platelet storage buffers has also been tested by others. Storing platelets in PAS containing up to 6 mM Mg^2+^ resulted in relatively minor changes in platelet metabolism (platelet count, pH, lactate concentration)^30^. Storage of platelets in PAS containing up to 2 mM Mg^2+^ resulted in higher platelet quality and reduced cytokine release in vitro^31,32^. A storage medium containing adenine, lidocaine, and 7 mM Mg^2+^ in PAS or plasma stored platelets preserved the aggregation response to ADP and TRAP-6 at RT and 4 °C storage^33^. In agreement with these findings, we observed that the aggregation response of platelets to collagen in PAS + with 30% plasma was better preserved during storage of 7 days at 4 °C in the presence of increasing concentrations of Mg^2+^.

Recently, we observed that cold storage also triggers phosphatidylserine exposure indicating platelet apoptosis^2^. Interestingly, Getz et al. observed that platelet apoptosis is reduced in citrate-free PAS^34^. Our results suggest that this might be due to increasing available Mg^2+^ by the absence of chelating agent citrate.

Cold storage of platelets also triggers an increased activation of metalloproteinases such as ADAMTS17, which in turn cleaves sialic acid from and GPIb leading to enhanced recognition and clearance of platelets by hepatic macrophages. ADAMTS17 inhibitors such as GM6001 block this effect in vitro^35^. During platelet storage also caspase enzymes are expressed and activated. They cleave numerous cellular proteins leading to cell death. Accordingly, it has been shown that Caspase-3 inhibitors block apoptosis during storage^36^.

In view of these other effects of cold storage induced platelet lesions, likely a combination of different approaches is required to preserve platelet in vivo recovery, survival, and function after cold storage of platelet concentrates.

Here we have shown that increasing Mg^2+^ concentrations to up to 10 mM is one important and easy to apply approach towards preventing cold-induced cytoskeleton storage lesions in platelets.

## Methods

### Platelet concentrates

Whole blood was collected from healthy donors according to the German guidelines for hemotherapy with written informed consent. The study was approved by the ethical board of the Universitätsmedizin Greifswald, Germany. All methods used were performed in accordance with the relevant guidelines and regulations. Pooled platelet concentrates from whole blood buffy coats were produced according to the standard method for producing therapeutic platelet concentrates. In brief, after centrifugation (4000×*g*, 10 min), whole blood in citrate phosphate dextrose solution (CPD, Macopharma, France) was separated into red cell concentrate, plasma, and buffy coat. Buffy coats of 4 blood group identical donations were pooled, and 250 mL additive solution PAS-E (SSP + , Macopharma) was added. PAS-E itself contains 2 mM magnesium. By centrifugation (720 g, 15 min), platelets were separated from residual red blood cells and leukocyte depleted (LEUCOFLEX**®** LXT Filter, Macopharma). PC bags were split into six bags (gas permeable bags, Macopharma) and stored under agitation at RT or 4 °C for 7 days.

### Platelet concentrate storage

Magnesium sulfate solution (Inresa Arzneimittel GmbH, Germany) was added to four of the six bags in increasing concentrations. 0, 2, 4, 6, 8 mM (final concentrations: 2, 4, 6, 8, 10 mM). PC was stored under agitation on an orbital shaker (LPR1, Melco Engineering Corp., USA). Sampling took place on day 0 (production day), day 1, day 4 and day 7.

### In vitro platelet function testing

The ability of platelets to respond to a hypotonic environment was determined as hypotonic shock response (HSR) by light transmission aggregometry (LTA)^37^. Platelets were exposed to distilled water or 0.9% w/v sodium chloride solution as a control. Due to the osmotic gradient, the water diffuses into the platelets, which leads to their swelling. As a result of swelling, the refractive index of platelets increases, resulting in a decrease in light transmission. The percentage of HSR was calculated as (T2 − T3)/(T2 − T1) × 100, with T1 = transmission of platelet suspension in sodium chloride; T2 = transmission of platelets in distilled water; T3 = “plateau” transmission value after 4 min of platelets incubated in distilled water.

Platelet activation was determined by CD62P expression before and after the addition of thrombin receptor activating peptide 6 (TRAP-6). 3 × 10^8^/mL platelets were incubated for 15 min at 37 °C with 20 µM TRAP-6 (Hart Biologicals, UK) or PBS buffer (w/o Ca^2+^, Mg^2+^; pH 7.2) as the negative control, fixed 20 min with 0.5% paraformaldehyde (Morphisto Laborchemikalien, Germany) and washed twice (2 mL PBS; 650 g, 7 min, RT). The pellet was resuspended in 500 µL PBS and analyzed by flow cytometry (FC500, Beckman Coulter, USA). Platelets were gated using a mouse-anti-human CD41-PeCy5 (clone P2, Beckman Coulter) labeled monoclonal antibody. The increase of CD62P exposure on CD41-positive events was determined using CD62P-FITC (clone CLBThromb/6, Beckman Coulter, USA). The mean fluorescence intensity of TRAP-6 activated platelets is given as fold increase in comparison to the respective buffer controls.

Platelet aggregation was analyzed over 7 min at 37 °C in a 4-channel-aggregometer (DiaSys, Germany) after the addition of 8 μg/mL collagen (Mölab, Germany).

For all in vitro platelet function analyzes we analyzed six pooled PC, in summary platelets from n = 24 different donors.

### Whole blood as a matrix for ex vivo quality control of platelet concentrates

To simulate in vivo conditions, we prepared platelet-depleted whole blood (dWB). Whole blood was collected from a healthy donor blood group O according to the German guidelines for hemotherapy, using a bag system containing citrate–phosphate-derivative with adenine (CPDA) solution for anticoagulation. Directly after donation, whole blood was leukocyte and platelet depleted by a LEUCOFLEX**®** LXT leukocyte depletion filter, which removes 99.999% of leukocytes and platelets (Macopharma, France). Cell counts were determined before and after filtration by an automated blood cell analyzer (Sysmex XP300, Sysmex Deutschland GmbH, Germany). Depleted whole blood was stored at 4 °C until use. Platelets stored at 4 °C at different Mg^2+^ concentrations were either analyzed directly in platelet storage buffer PAS-E or spiked into depleted whole blood to achieve a final platelet concentration of 300,000/µL and rewarmed at 37 °C for 1 h before analyses. For aggregometry studies, whole blood was centrifuged at 120×*g* for 20 min to obtain PRP.

### Biomechanical platelet characterization

RT-DC is a method for the biomechanical characterization of cells with throughput rates of up to 1,000 per second (Supplementary Fig. S2)^23^. In brief, platelets are pumped at flow rates of 0.006 μL/s from a reservoir through a narrow channel on a chip assembled on the AcCellerator system (Zellmechanik Dresden). The microfluidic chip consists of a constriction of 15 μm × 15 μm cross-section with a length of 300 μm and is connected to a syringe pump (NemeSys, Cetoni, Germany). Upon entering the channel and during the passage, the cells are subjected to both hydrodynamic shear forces and different pressure gradients. The deformation of platelets caused by these external forces are recorded by a high-speed camera. The individual images are then analyzed with an evaluation algorithm (ShapeOut, version 0.8.6, Zellmechanik Dresden) for the deformation of the individual platelet (Supplementary Fig. S2)^17^. Samples of n = 6 pooled PC were taken on days 1, 4, and 7 after PC production. For RT-DC 50 μL of platelets in PAS-E/plasma were diluted in Carrier B (Zellmechanik Dresden; 0.6% (w/v) methylcellulose in PBS, without Ca^2+^ and Mg^2+^) to a final concentration of approximately 1 × 10^7^/mL. Calculations are performed at different flow rates representing different experimental conditions.

### Analysis of cytoskeleton proteins by immunofluorescence microscopy

Platelets from PC of all 6 storage conditions were adjusted to a platelet count of 50,000 platelets/µL each with 2% paraformaldehyde in PBS (w/o Ca^2+^, Mg^2+^, pH 7.2) and incubated for 15 min at RT. At the end of the incubation period, 100 µL of each sample was cytospin centrifuged by for 5 min at 700 rpm on microscopic slides. Following centrifugation, the slides were washed three times for 5 min at RT with PBS (w/o Ca^2+^, Mg^2+^, pH 7.2) and stored at − 20 °C. The cells were treated with permeability buffer (0.1% Triton-X in PBS and BSA 2%) for 10 min at RT in a humid chamber, and cells were then washed twice with 50 µL PBS for 10 min at RT. Platelets were stained for α-tubulin (mouse anti-human α-tubulin IgG, Sigma-Aldrich, St. Louis, Missouri, USA) at RT for at least 1 h, washed again twice with 50 µL PBS and then incubated with 50 µL secondary donkey anti-mouse IgG Alexa Flour 568 (Abcam, Cambridge, UK) for 1 h at RT, incubated with 50 µL of phalloidin ATTO 488 solution (1:200; ATTO-TEC GmbH, Siegen, Germany) for further 30 min at RT in the dark and then washed twice with PBS. Fluorescence microscopy was performed on a Leica SP5 confocal laser scanning microscope (Leica Microsystems, Wetzlar, Germany) equipped with HCX PL APO lambda blue 40.0x/1.25 OIL UV objective. For image acquisition, fluorescent tags Atto-488 and AlexaFluor-568 were excited by argon (488 nm) and helium–neon (HeNe) 563 nm laser lines, respectively, selected with an acousto-optic tunable filter (AOTF). Fluorescence emission for Atto-488 and AlexaFluor-568 was collected between 505–515 nm and 550–570 nm on hybrid detectors (HyD) and photomultiplier tubes (PMTs), respectively. Assessment of distribution and organization of marginal band α-tubulin staining was performed by measuring cross-sectional line profile (5 µm length and 1 µm width) of non-saturated grayscale fluorescence intensities (pixel values) of immunofluorescent probes across individual platelets in confocal images using Leica Application Suite X (Version 3.7.1, Leica Microsystems, Wetzlar, Germany). Data were plotted using GraphPad Prism version 8.0.0 for Windows, (GraphPad Software, San Diego, California USA). For each storage condition, the microscopic images of at least 10 single platelets were analyzed. In addition, whole blood spiked with platelets from stored PC was also used to prepare blood smears, which were stained as described above. Here, an Olympus BX 40 microscope with UPlanSApo 60x/1.35 Oil objective was used with software cellSensStandard (Imaging Software cellSens, Olympus Corporation, Tokyo, Japan).

### Desialylation of stored platelets

To evaluate the effect of Mg addition on desialylation of platelets during storage at 4 °C we determined the binding of fluorescein isothiocyanate (FITC) labeled *Erythrina cristagalli* lectin (ECL) and *Ricinus communis* lectin agglutinin (RCA) to platelet surface glycoproteins of RT and cold stored platelets (n = 3 pooled PC) with or without Mg^2+^ addition. PC was adjusted to a cell count of 300,000/µL and labeled by CD61-AlexaFluore647 platelet marker (Biolegend, USA). FITC labeled RCA and ECL were added (1 µL 1:500 RCA or 1:250 ECL in PAS-E, Biozol, USA) and incubated in the dark for 20 min. After that 200 µL PAS-E was added, and the samples were washed (650×*g*, 7 min). The binding of ECL and RCA was analyzed using an FC500 flow cytometer (Beckman Coulter, USA). As a positive control, sialidase (neuraminidase, Sigma Aldrich, USA) was used.

### Statistical data evaluation

For all statistical data evaluation we used Graphpad Prism 8.0.1. Statistical significances were calculated by One-Way Analysis of Variance (ANOVA) with multiple comparisons test.

## Supplementary Information

Supplementary Information.

## Data Availability

The datasets generated during and/or analysed during the current study are available from the corresponding author on reasonable request. All data generated or analysed during this study are included in this published article (and its Supplementary Information files).
