# Supplementary material for: Divalent magnesium restores cytoskeletal storage lesions in cold-stored platelet concentrates
Source: Sci Rep. 2022 Apr 14;12:6229. doi: 10.1038/s41598-022-10231-x (PMC9010418; doi:10.1038/s41598-022-10231-x)
Supplement: Supplementary file 1 — Supplementary Information. [file 41598_2022_10231_MOESM1_ESM.pdf]

Divalent magnesium restores cytoskeletal storage lesions in cold-stored platelet concentrates

Konstanze Aurich, Jan Wesche, Martin Ulbricht, Oliver Otto, Andreas Greinacher, Raghavendra Palankar

Supplementary Figures

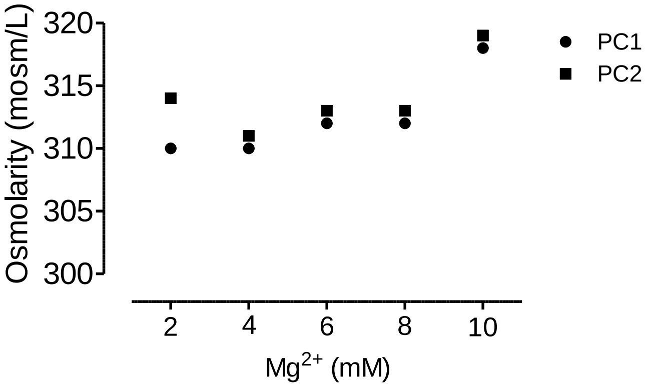

Supplementary Figure S1. Total osmolarity of two platelet concentrates after Mg^{2+} addition

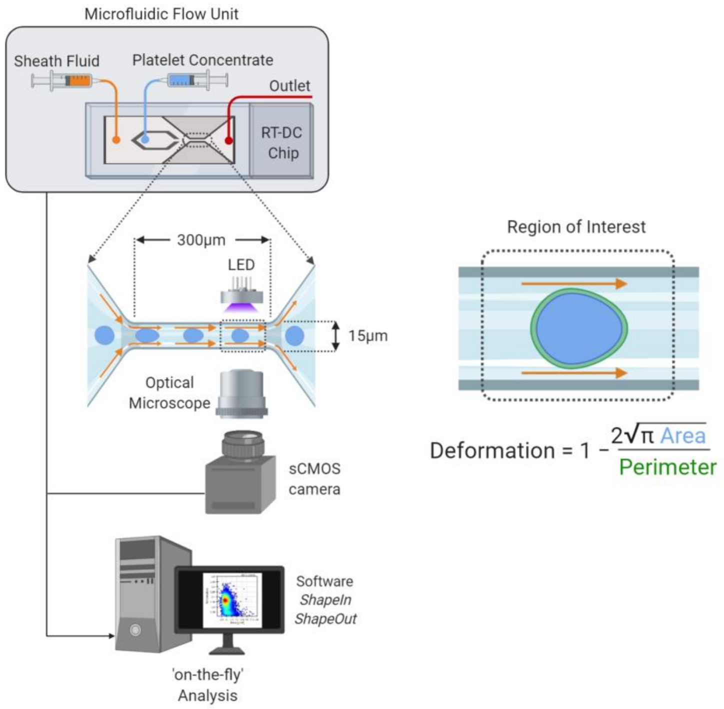

Supplementary figure S2. Real-time fluorescence and deformability cytometry. (A) The setup consists of a microfluidic chip, with a central constriction of 300 µm length and 15 µm × 15 µm cross-section, where cells in the sample fluid are surrounded by a sheath fluid and deformed by shear and normal forces where cell/platelets undergo deformation as a result of hydrodynamic compression brought about by sheath fluid. A high-powered light-emitting diode (LED) is used to illuminate the samples. Images are recorded by a scientific complementary metal–oxide–semiconductor (sCMOS) camera working at high frame rates (≈2000 images/s) that is synchronized with the frequency of illumination time. This combined together with microfluidic flow, RT-DC allows for mechanoprofiling of >100 unique cells/platelets per second in a contact- and label-free manner. (B) Deformation of cells is calculated from the circularity using ratio of cell area and perimeter.
